# An unsupervised automatic segmentation algorithm for breast tissue classification of dedicated breast computed tomography images

**DOI:** 10.1002/mp.12920

**Published:** 2018-05-09

**Authors:** Marco Caballo, John M. Boone, Ritse Mann, Ioannis Sechopoulos

**Affiliations:** ^1^ Department of Radiology and Nuclear Medicine Radboud University Medical Center PO Box 9101 6500 HB Nijmegen The Netherlands; ^2^ Department of Radiology and Biomedical Engineering University of California Davis Health 4860 “Y” street, suite 3100 Ellison building Sacramento CA 95817 USA; ^3^ Dutch Expert Center for Screening (LRCB) PO Box 6873 6503 GJ Nijmegen The Netherlands

**Keywords:** breast cancer, CAD, dedicated breast CT, image classification, segmentation

## Abstract

**Purpose:**

To develop and evaluate a new automatic classification algorithm to identify voxels containing skin, vasculature, adipose, and fibroglandular tissue in dedicated breast CT images.

**Methods:**

The proposed algorithm combines intensity‐ and region‐based segmentation methods with energy minimizing splines and unsupervised data mining approaches for classifying and segmenting the different tissue types. Breast skin segmentation is achieved by a region‐growing method which uses constraints from the previously extracted skin centerline to add robustness to the model and to reduce the false positive rate. An energy minimizing active contour model is then used to classify adipose tissue voxels by including gradient flow and region‐based features. Finally, blood vessels are separated from fibroglandular tissue by a k‐means clustering algorithm based on automatically extracted shape‐based features. To evaluate the accuracy of the algorithm, two sets of 15 different patient breast CT scans, each acquired with different breast CT systems and acquisition settings were obtained. Three slices from each scan were manually segmented under the supervision of an experienced breast radiologist and considered the gold standard. Comparisons with manual segmentation were quantified using five similarity metrics: Dice similarity coefficient (DSC), sensitivity, conformity coefficient, and two Hausdorff distance measures. To evaluate the robustness to image noise, the segmentation was repeated after separately adding Gaussian noise with increasing standard deviation (in four steps, from 0.01 to 0.04) to an additional 15 slices from the first dataset. In addition, to evaluate vasculature classification, three different pre‐ and postcontrast injection patient breast CT images were classified and compared. Finally, DSC was also used for quantitative comparisons with previously proposed approaches for breast CT tissue classification using 10 images from the first dataset.

**Results:**

The algorithm showed a high accuracy in classifying the different tissue types for both breast CT systems, with an average DSC of 95% and 90% for the first and second image dataset, respectively. Furthermore, it demonstrated to be robust to image noise with a robustness to image noise of 85%, 83%, 79%, and 71% for the images corrupted with the four increasing noise levels. Previous methods for breast tissues classification resulted, for the tested dataset, in an average global DSC of 87%, while our approach resulted in a global average DSC of 94.5%.

**Conclusions:**

The proposed algorithm resulted in accurate and robust breast tissue classification, with no prior training or threshold setting. Potential applications include breast density quantification and tissue pattern characterization (both biomarkers of cancer development), simulation‐based radiation dose analysis, and patient data‐based phantom design, which could be used for further breast imaging research.

## 1 Introduction

Dedicated breast computed tomography (breast CT) is a recent imaging modality that can provide real three‐dimensional (3D) images of the breast with high spatial and contrast resolution. Although conventional computed tomography has been extensively utilized in clinical studies, it is not optimized for breast imaging, and is hardly ever used for this purpose.^1^ Dedicated breast CT, optimized for the special contrast, spatial resolution and tissue coverage requirements of breast imaging, might bring the advantages from CT as seen in body imaging to breast imaging.^2^ Compared to mammography and digital breast tomosynthesis, breast CT, beside completely avoiding tissue superimposition, has the additional advantage of imaging the breast without the need for compression, increasing patient comfort.

Given the full 3D nature of dedicated breast CT and therefore the possibility to isotropically image the entire organ, complete breast tissue characterization can be achieved and several biomarkers for cancer development may be evaluated.^3^ For instance, breast density is a relevant breast cancer risk factor.^4^ In addition, staging of known breast cancer may be improved by automatically assessing changes in the normal structures. For example, breast lymphoma and inflammatory breast cancer (as well as certain types of metastases) may show skin thickening.^5^ Finally, literature reports a relationship between vascular maturation and breast cancer. In neoplastic breasts, a remodeling of blood vessels may occur, due to the proliferation of endothelial cells which is strictly related to angiogenesis.^6^

Classification of breast tissue can therefore provide quantitative assessments of breast tissue composition, density, and distribution that can be used to evaluate the risk of breast cancer and guide therapy.^7^

Pike et al.^8^ proposed a minimum spanning forest‐based method for breast tissue classification, while the approach followed by Nelson et al.^9^ for segmentation of breast CT images is based on the image histogram. Yang et al.^10^ developed a classification algorithm for high‐resolution breast CT based on bilateral filtering able to segment skin, adipose, and glandular tissue within the breast. Density‐based breast classification has also been explored in mammography in an effort to create a rating for breast tissue composition.^11^ Finally, in the work of Huang et al.,^12^ a radial‐geometry edge detection scheme to measure the breast skin thickness on coronal reconstructed breast CT images was proposed.

In this study, for a complete breast characterization, we focus on the classification of all major tissue types within the breast, which include skin, fibroglandular tissue, adipose tissue, and vasculature. The proposed fully automatic algorithm combines intensity‐ and region‐based segmentation methods with energy minimizing splines and unsupervised data mining approaches to classify the breast tissues with no prior training or threshold setting.

In some previous approaches, region‐based segmentation (which we used to classify the voxels belonging to the skin) was obtained via region‐growing based on an intensity threshold, with manually selected seeds.^13^ Others, to reduce the number of mis‐segmented voxels due to threshold setting, propose growth‐limiting criteria^14^ or, alternatively, dual object and background competitive region‐growing methods.^15^ These previous approaches assume voxels with similar intensity levels belonging to the same region (a criterion that could match well the initial conditions of their segmentations), while our segmentation scheme is aimed at avoiding the inclusion of tissue structures adjacent to the inner skin boundary that have similar HU values (e.g., blood vessels). Therefore, for our application, a different approach was needed to include distance‐based constraints which can reduce the number of false positives.

Regarding the energy minimization problem (used to separate the adipose from fibroglandular tissue and vasculature), many implementations of energy minimizing active contours have been proposed in literature. Broadly, they can be divided into two main classes: edge‐based techniques and region‐based approaches. Among the edge‐based techniques, which mostly rely on the intensity of image edges to guide the evolution of the contour,^16^ two well‐known models are geo‐cuts and geodesic active contours. The former are usually able to provide a globally optimal solution in low‐order polynomial time, but the accuracy of the segmentation is often strongly dependent on constraints and markers which have to be defined in advance.^17^ The latter, based on the search of the minimal image geodesic energy, do not require any markers, but are very sensitive to image local minima, i.e., they could converge even if the global optimal solution is not reached.^18^ So as to overcome this problem, the evolution equation can be modified in order to jump over shallow local minima (e.g., by adding balloon forces), but this often results in additional user‐selectable parameters.^19^ Region‐based active contours can be broadly divided into two main groups: active contours without edges (mostly based on Chan‐Vese model) and localizing region‐based active contours. The former use an evolving curve to separate the image domain in two regions with smooth boundaries and minimal intraclass variance.^20^ In their original form, in each region, a constant gray‐value is supposed to approximate the image, and they usually include weights to overcome large differences in intensity within the same region.^20^ To improve these models (which depend on a few assumptions), localizing region‐based active contours have been developed. The idea is to restrict the region energy computation to a small neighborhood of contour points using a characteristic function.^21^ This allows for a better evaluation of the image in different spatial positions, although localization often leads to higher sensitivity to initialization.^21^ In this work, we implemented a different energy minimizing contour model matching both edges and region‐based image features.

The effectiveness of the whole algorithm we propose comes from its ability to effectively incorporate different image analysis techniques, each suitable for classification of a different tissue type. Furthermore, the incorporation of breast blood vessels segmentation, which has not been previously proposed in literature, adds accuracy to the algorithm. The robustness and performance of the proposed algorithm were demonstrated with real patient data.

The possibility to image the entire organ in three dimensions with high resolution using dedicated breast CT could provide further insight regarding organ composition in terms of tissue types. Moreover, the possibility to automatically segment these tissues may allow researchers to fulfill several goals which would not be achievable without the full third dimension and without tissue classification. As stated above, correlation between certain tissue patterns and cancer development could be searched so as to provide more accurate results compared to the studies already published in literature (which, to our knowledge, have been mainly conducted on mammography^4^, ^5^). Furthermore, tissue classification could be very important to accurately assess radiation dose guidelines, since patterns and quantification of fibroglandular tissue from real patient data could be evaluated through radiation‐based simulation software tools.

## 2 Materials and methods

### 2.A. Equipment and image acquisition

Patient images were acquired with two different breast CT systems. The first patient dataset was acquired with a breast CT system^22^, ^23^, ^24^ (Koning Corp., West Henrietta, NY, USA) installed at Radboud University Medical Center. The x‐ray tube with a tungsten target and aluminum filter was set to a voltage of 49 kV, resulting in an x‐ray spectrum with a first half value layer of 1.39 mm Al with a 0.3 mm nominal focal spot. The detector was 397 mm × 298 mm (4030CB, Varian Medical Systems, Palo Alto, CA, USA), resulting in a reconstructed voxel size of 273 μm (the reconstruction algorithm used was Filtered Back Projection). The source‐to‐imager distance of the breast CT system was 92.3 cm, while the source‐to‐isocenter distance was 65 cm.

Images were acquired with the x‐ray tube operating in pulsed mode, with a constant 8 ms pulse, and the tube current for each patient breast automatically set by prior acquisition of two scout images normal to each other (16 mA, 2 pulses of 8 ms each per projection). According to the signal level inside the two breast scouts, a tube current between 12 and 100 mA was selected for acquisition of the breast CT scan. A complete breast CT scan involved the acquisition of 300 projections over a full 360° revolution of the x‐ray tube and detector in 10 s. The dose varied for each patient breast, with the average value for a breast of mean size and composition being 17 mGy.^24^

The second system used for patient image acquisition is a third‐generation dedicated breast CT prototype^25^ installed at University of California, Davis (USA). The gantry design includes a pulsed x‐ray tube (M‐1500, Varian Medical Systems, Salt Lake City, UT, USA) set to a voltage of 60 kV (nominal focal spot size of 0.3 mm) with an added 0.2 mm Cu filter, resulting in a first half value layer of 1.5 mm Al. The system uses a flat‐panel detector which features a 0.45 mm thick thallium‐activated structured cesium iodide (CsI:Tl) scintillator coupled to complementary metal oxide semiconductor (CMOS) active detector elements sensors (DEXELA 2923MAM, Perkin Elmer, Santa Clara, CA, USA). The detector provides an active field of view of 290 mm × 230 mm. The voxel size is not fixed and can vary from (0.194–0.407)^2^ × 0.210 mm while the reconstruction algorithms consist of a variation in the Feldkamp algorithm.^26^ Images from this system are acquired with a tube current of 240 mA and a constant 10.8 ms pulse is used. A complete breast CT scan was composed of 285 projections, and the average dose was approximately 5 mGy.

All images were acquired by trained radiographers, as part of other ethics board‐approved patient trials on dedicated breast CT, and all subjects provided written informed consent.

### 2.B. Classification algorithm

**Figure 1:**
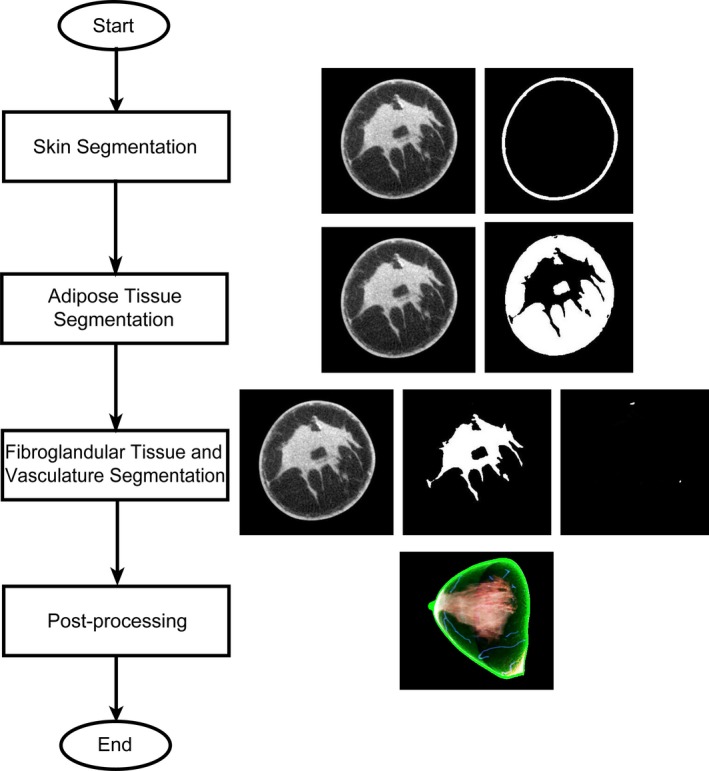
Schematic representation of the processing steps of the algorithm. [Color figure can be viewed at http://wileyonlinelibrary.com]

The algorithm is composed of the following steps: an intensity and distance‐based segmentation method for breast skin detection; energy minimizing splines for adipose tissue segmentation; an unsupervised pattern recognition approach to classify the remaining tissue types into fibroglandular tissue and vasculature. The entire pipeline of the algorithm in shown in Fig. 1. The algorithm works in a slice‐by‐slice manner, subsequently considering one tissue type at a time. After evaluating all slices, a continuity criterion to refine blood vessel classification is applied as a postprocessing step. The images were processed using a 2.4 GHz CPU, 12 GB RAM workstation.

#### 2.B.1. Breast skin segmentation

The first step of the algorithm is to automatically detect and classify the skin of the breast. For this, first the outer edge of the skin is identified using a Sobel gradient. Second, the centerline of the skin is detected. Several methods for centerline extraction have been previously proposed.^27^, ^28^, ^29^ The algorithm developed in this work is partly based on the intensity of Height Ridge Transversal and multiscale extraction.^30^

The centerline algorithm starts with the search of candidate points, named seeds, belonging to the external edge of the skin. The eight‐connected neighborhood of each seed point is taken into account and the search of the ridge is performed along the line that joins the seed point and the maximum intensity voxel in the neighborhood. If the intensity difference between the maximum intensity voxel of the neighborhood and the seed point is higher than 0, the direction is saved for the following iteration, otherwise the seed point is defined as a one‐dimensional (1D) maximum candidate and the search ends. This operation allows to get closer to the ridge (i.e., the central part of the skin, where the HU values are usually higher). Moreover, performing this search along a line (i.e., in one dimension) reduces the computational time considerably. All the 1D maximum voxels are required to meet two criteria in order to be considered ridge points, i.e., part of the centerline. Since the algorithm is specifically designed to search maximum convexity height ridges, the basis directions normal to a ridge are defined using eigenvalues and eigenvectors of the Hessian matrix of the image. Hessian eigenvalues ($\lambda_{1}$, $\lambda_{2}$) and associated eigenvectors (${\vec{v}}_{1}$, ${\vec{v}}_{2}$) are sorted so that:

(1) $$\left|\lambda_{1_{\left(i,j\right)}}\right|⩽\left|\lambda_{2_{\left(i,j\right)}}\right|$$

where (i, j) are the coordinates of a given voxel (row and column) on the considered reconstructed slice.

In particular, a point is labeled as a 1D ridge of a two‐dimensional (2D) surface if:

1. N‐1 of the eigenvectors of the Hessian matrix of the image have negative eigenvalues. This condition is tested by ordering the eigenvalues and verifying that

(2) $$\lambda_{1_{\left(i,j\right)}}<0$$

1. The point belongs to an (N‐1)‐dimensional extreme, i.e., the projection of the image gradient *∇I* at the considered point onto the N‐1 directions normal to the ridge is equal to zero:

(3) $${\vec{v}}_{1}\cdot \nabla I=0$$

In practice, this condition is met if the previous equation is lower than a given threshold (*tolerance‐to‐zero threshold*), which we set to 10^−6^.

**Figure 2:**
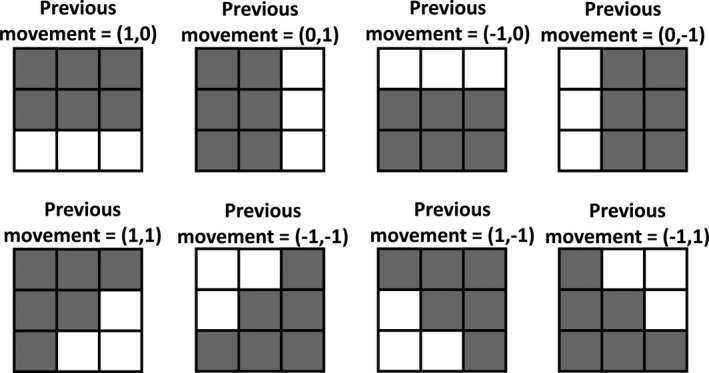
2D eight‐connected kernels of the forbidden direction of search (in gray) for the centerline algorithm. The vector of (x, y) positions indicates the direction of search of the previous iteration.

**Figure 3:**
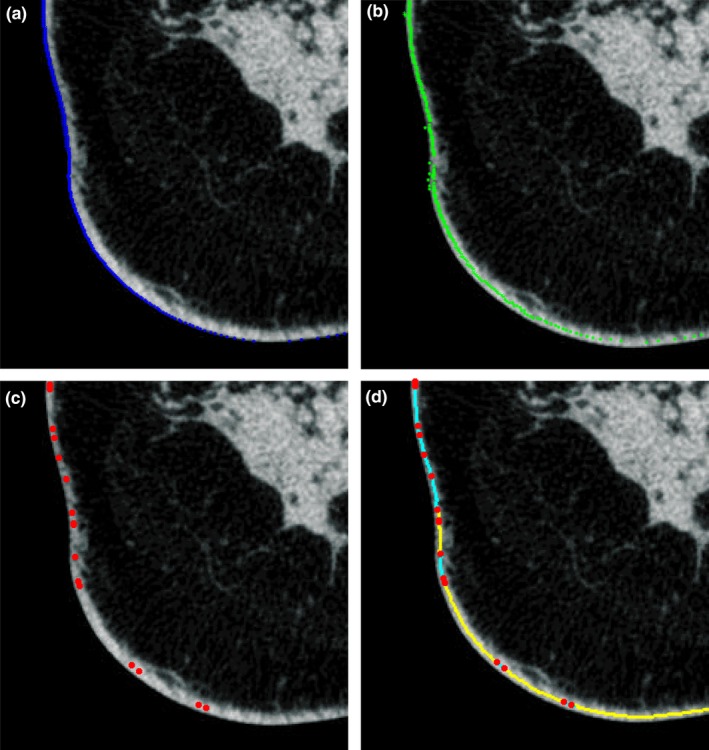
Main steps of the centerline algorithm. (a) representation of initial seed points; (b) identification of the 1D maximum candidates; (c) selection of the ridge points; and (d) forward and backward path of search. [Color figure can be viewed at http://wileyonlinelibrary.com]

After all the ridge points are detected, an intensity‐based search of the centerline is performed. Starting from the first ridge point, the maximum intensity voxel in its eight‐connected neighborhood is labeled as the first voxel of the centerline and defines the forward path of search. From this new voxel, another maximum intensity voxel is identified in its neighborhood and the process is iteratively repeated along the forward path. In each step of this search, some positions of the eight‐connected neighborhood are forbidden according to the previous iteration (see Fig. 2 for details). This process is needed so as not to allow the centerline to deviate from its central path in case of high discontinuities in voxel HU. The search of the forward path ends when all starting ridge points are evaluated. When the forward path of search ends, the search is repeated, starting from the first voxel opposite of the first point found near to the considered ridge point. The search continues in the opposite direction, along the backward path of search, following the same criteria of the forward path of search and ending when all ridge points are linked together (Fig. 3).

After the complete centerline is extracted, a region‐growing algorithm based on double thresholding is used to segment the skin. Here, the two thresholds are given by the average values of the voxels of the external edge (the lowest threshold) and of the computed centerline (the highest threshold), which also defines the set of seeds for the region growing. Moreover, the highest distance (*d*) between the external edge and the centerline is calculated. Starting to grow the region from the seeds, a voxel *P* is classified as breast skin if its gray level lays between the two thresholds and if the closest Euclidean distance between *P* and the centerline is shorter than (or equal to) *d*. This latter criterion is very useful to avoid the segmentation of other structures (such as fibroglandular tissue or blood vessels) whose intensities are similar to the skin and which can be adjacent to it. The region growing stops when no further voxels fulfill the previously stated criteria.

#### 2.B.2. Adipose tissue segmentation

After the skin is segmented, an energy minimizing active contour model is used to automatically identify blood vessels and fibroglandular tissue (which will be temporarily included within the same class). Adipose tissue voxel classification is then obtained by subtracting the result of the active contour segmentation from the skin‐free breast CT slice.

The contour is automatically initialized with a voxel within the fibroglandular tissue. Although the contour does not strictly depend on initialization (i.e., it could be initialized either on fibroglandular tissue or on vasculature), automatic initialization within fibroglandular tissue is easier to perform due to its large size compared to blood vessels. In most cases, the voxel with the maximum intensity in the image corresponds to fibroglandular tissue. However, in some cases, calcifications, metal clips, implants, or some artifact could have higher HU voxel values. Thus, an approach based on the image histogram was developed to initialize the active contour. The image histogram is computed once the previously segmented skin has been removed. Since the HU values for fibroglandular tissue voxels differ considerably from those of adipose tissue, the histogram shows a bimodal distribution. A threshold is chosen by taking the minimum histogram value from the valley that separates the two distributions to create a binary mask. The choice of this threshold is as follows. After excluding the background voxels, the image histogram is smoothed using a moving average filter with a window size of 9 (i.e., the number of points used to average the input histogram), and the two highest maxima (i.e., the ones of the two distributions) are identified using morphological reconstruction. After finding the maxima, the minimum is found by taking the lowest value between these two maxima. The binary mask obtained from this process is a very rough initial partition of the image in two classes: one for the adipose tissue (and a very few low‐intensity blood vessels) and the other which includes the fibroglandular tissue and the majority of blood vessels. This partition cannot be considered accurate (especially due to the HU variations of each tissue type); therefore, it is only used to correctly initialize the active contour, and not as a reliable tissue classification. Connected components within the slice from the latter class are identified and their areas are calculated. Since blood vessels present smaller areas on the binary mask compared to that of the fibroglandular tissue, a voxel within the largest object is selected and the active contour is initialized around that voxel.

In this work, we propose a new energy minimizing active contour model to segment the breast fibroglandular tissue and blood vessels by including both region‐ and edge‐based information. The model for the segmentation framework comes from Bayesian inference,^31^ that is, searching for the contour shape that maximizes the posterior probability by minimizing an energy function. The image energy of the proposed model is derived from the image gradient feature, which gives the active contour a global representation of the geometric configuration (internal force), and it is driven by the local variation in image intensity features (external force).

The energy function that has to be minimized is described as follows:

(4) $$E\left(t\right)=\int_{\Omega_{\mathrm{ext}}}{\left(\sigma \left(s\right)-k\right)}^{2}ds+\frac{\partial \sigma_{\mathrm{int}}}{\partial t}+\left[\frac{\partial I_{\mathrm{int}}}{\partial t}\right]\int_{\Omega_{\mathrm{int}}}I{\left(s\right)}^{2}ds+\left[\frac{\partial I_{\mathrm{ext}}}{\partial t}\right]\int_{\Omega_{\mathrm{ext}}}I{\left(s\right)}^{2}ds+\int_{0}^{1}{\left|\nabla I(C\left(s\right))\right|}^{-1}ds$$

At each iteration (*t*), several image parameters inside ($\left(\Omega_{\mathrm{int}}\right)$, outside ($\left(\Omega_{\mathrm{ext}}\right)$, and along the active contour (*C*) are calculated as shown in Eq. (4). Each term is supposed to be null (or at least reach its minimum value) when convergence is reached. The combination of these terms is designed to be robust to image noise and to easily avoid image local minima, with no need to set any user‐selectable parameters.

In detail, the variance inside the active contour ($\sigma_{\mathrm{int}}$) tends to stabilize after a sufficiently large number of iterations due to the increase of the amount of pixels inside the contour. Therefore, its first derivative tends to become null. The same is true for both the mean intensity value inside ($\underset{\mathrm{int}}{I}$) and outside ($\underset{\mathrm{ext}}{I}$) the contour.

Using the first derivative instead of the primitive values (mean and standard deviation) makes these contributions parameter‐free, since no weights are needed so as to take into account intensity variations within the same region.

The image variance outside the contour, *σ(s)*, tends to decrease at each iteration, but it does not always stabilize (mostly due to image noise). Therefore, a constant value (*k*) is subtracted from its contribution (given by its integral in the outer domain). The parameter *k* is automatically computed for each slice, and it gives an *a priori* estimation of the variance of the adipose tissue. To automatically calculate the parameter *k*, the lowest intensity pixel interior to the skin (which has been already classified) is identified, and a 30‐iteration intensity‐based region growing is performed starting from that pixel. The membership criterion to the region is based on two thresholds, both calculated from the image histogram previously computed during the active contour initialization. Respectively, they are given by the 90% and 10% of the minimum of the valley that separates the two histogram distributions. Once the active contour discriminates the adipose tissue from the other tissue types well, the difference between *σ(s)* and *k* tends to zero.

Finally, the inverse of the image gradient (${\left|\nabla I(C\left(s\right))\right|}^{-1}$), calculated at each iteration along the active contour *C* and which decreases as the contour gets closer to image edges, drives the curve toward the object boundaries.

At each iteration, the contour model, whose path is defined as a curve $C\left(s\right)=\left(x\left(s\right),y\left(s\right)\right)$ of the image domain, is updated through the following equation:

(5) $$C\left(t+1\right)=C\left(t\right)+E\left(t\right)\cdot \sqrt{{\left(\frac{dC(t)}{\mathrm{dx}}\right)}^{2}+{\left(\frac{dC(t)}{\mathrm{dy}}\right)}^{2}}$$

and spatially expanded through the convolution with a eight‐connected Gaussian kernel with standard deviation equal to 0.5.

**Figure 4:**
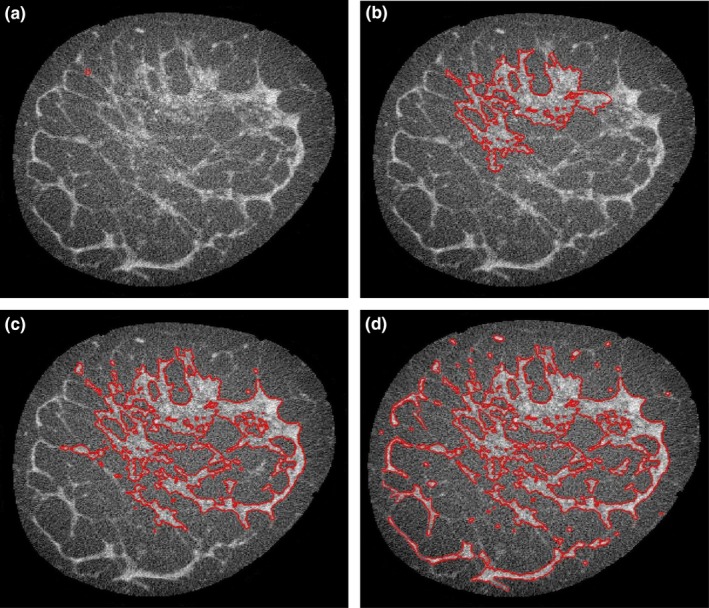
Active contour: (a) initialization; (b) after 70 iterations; (c) after 140 iterations; and (d) convergence. [Color figure can be viewed at http://wileyonlinelibrary.com]

**Figure 5:**
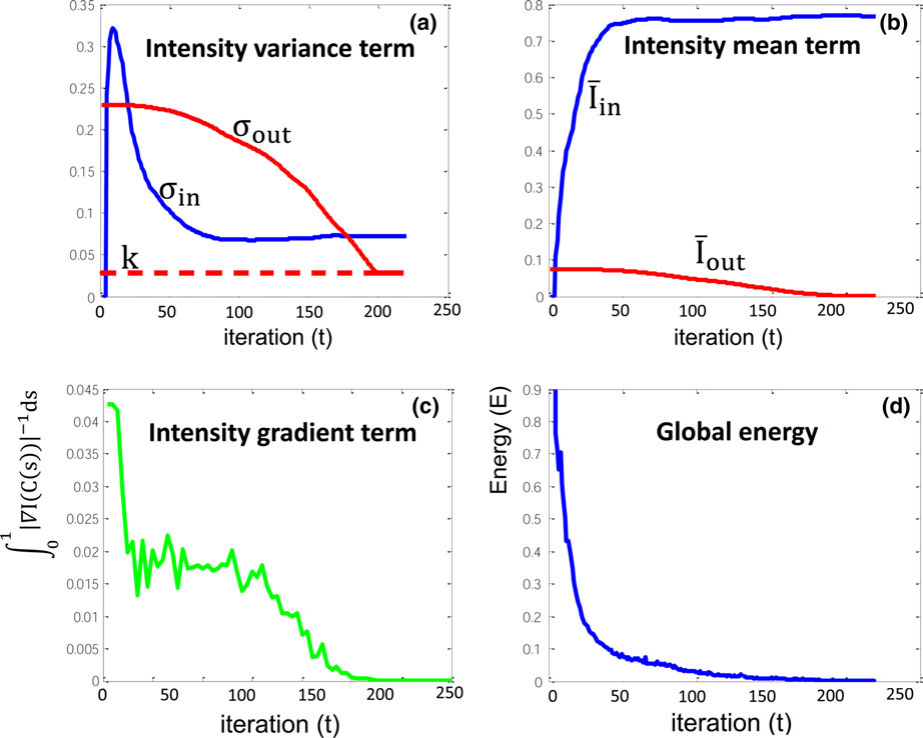
Example of the primitive values of energy contributions and global energy for the active contour model over the number of iterations: (a) the variance inside and outside the contour and the automatic estimation of the adipose variance; (b) the image mean values inside and outside the contour; (c) the inverse of the image gradient; and (d) the global energy. [Color figure can be viewed at http://wileyonlinelibrary.com]

If, at a given iteration, the contour does not expand anymore but the global energy is not yet null (this could happen due to edge forces), it gets dilated and propagated to close structures whose intensity is included in the range [${\overset{¯}{I}}_{\mathrm{int}}\pm \sigma_{\mathrm{int}}$]. This is useful to overcome local minima convergence while still keeping edge information, especially in the case of sparse fibroglandular tissue where structures may not be always fully connected within the same slice. This propagation is performed repeatedly until the global energy becomes null (due to discretization and image noise, we consider the energy to be null if it becomes lower than 10^−3^). An example of the active contour and its energy contributions are shown in Figs. 4 and 5, respectively. When the contour reaches the optimal solution, the resulting segmentation is subtracted from the slice, resulting in only adipose tissue remaining.

#### 2.B.3. Vasculature and fibroglandular tissue classification

At this point of the algorithm, two tissue types are yet to be distinguished from each other and classified: blood vessels and fibroglandular tissue. We applied a k‐means clustering algorithm^32^, ^33^ for the classification of these remaining tissues, after removing the already segmented voxels.

The clustering algorithm proposed in this work is based on intensity and geometrical features of each connected component identified within a slice. To avoid misclassification (due to the incorrect choice of the number of clusters) in those slices where only one tissue type is present, the feature extraction process and the subsequent cluster partition are applied (in a slice‐by‐slice manner) to the entire 3D breast CT image once the skin and the adipose tissue have been excluded from each slice. In other words, classification (as well as the feature extraction) is always performed slice‐by‐slice, but the feature space is derived from all the slices of the breast CT image.

Slice‐by‐slice, each remaining structure is labeled and four features (from each connected component) are extracted:

- HU variance
- Circularity

(6) $$C=\frac{4\pi \cdot Area}{{\left(\mathrm{Perimeter}\right)}^{2}}$$

- Aspect Ratio:

(7) $$AR=\left|\frac{\min(d)}{\max(d)}\right|$$

where *d* is the set of all possible distances between the center of mass of the structure and its boundary.

**Figure 6:**
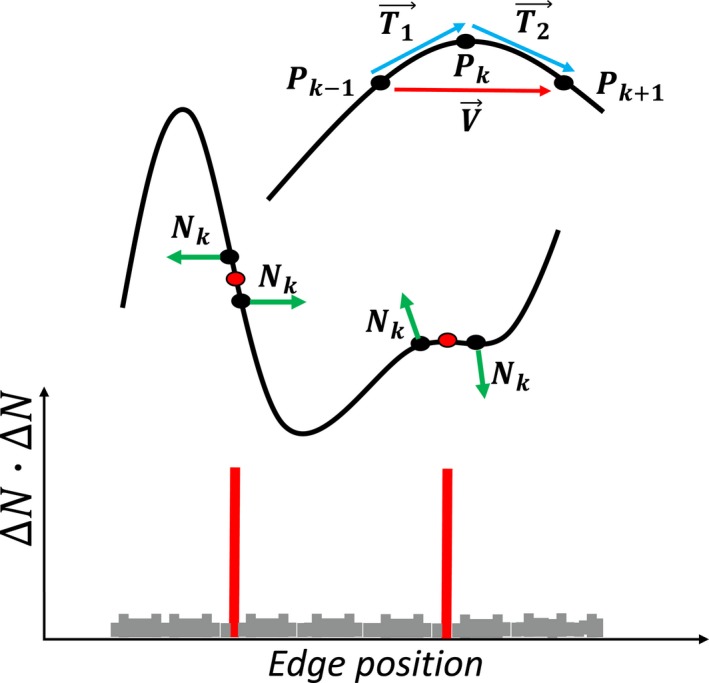
Schematic representation of inflection points computation. [Color figure can be viewed at http://wileyonlinelibrary.com]

- Number of boundary inflection points. A point along the boundary of the structure is considered an inflection point if:

(8) $$\vec{\Delta N}\cdot \vec{\Delta N}>1$$

where (see Fig. 6 for details):

(9) $$\vec{\Delta N}={\vec{N}}_{k+1}-{\vec{N}}_{k+1}$$

(10) $${\vec{N}}_{k}=\frac{\vec{V}\times \left({\vec{T}}_{2}-{\vec{T}}_{1}\right)\times \vec{V}}{\|\vec{V}\times \left({\vec{T}}_{2}-{\vec{T}}_{1}\right)\times \vec{V}\|}$$

From mathematics, in every inflection point, the second derivative of a curve (in our case, the object boundary) is equal to zero. Since, in image processing applications, an approach based on the computation of second derivative of an edge often leads to inaccuracy due to the fact that pixels are not dimension‐free,^34^ we adopted a more robust method which computes the normal vectors in each point of the edge.^35^ Across an ideal inflection point, the normal unit vector changes its direction by 180°. Therefore, the quantity $\vec{\Delta N}$ becomes equal to 2, and the scalar product $\vec{\Delta N}\cdot \vec{\Delta N}$ gives 4. This could be false for edges of a real image, in which each point is a pixel with finite dimensions. Therefore, in an attempt to not miss any point in which the edge changes concavity, we consider a boundary pixel an inflection point if the dot product is greater than 1.

After extracting the features, the clustering algorithm is applied and the dataset is divided into three classes: two for recognizing blood vessels and one for recognizing the fibroglandular tissue.

The need for two classes for the vasculature is based on the fact that blood vessels may appear, within one slice, approximately either circular or tubular, while fibroglandular tissue tends to show a much more irregular profile. In Table 1 typical qualitative values for these features are shown.

**Table 1:** Typical values for the four features used in fibroglandular tissue — blood vessels classification

	Circular structure	Tubular structure	Irregular structure
Circularity	HIGH	LOW	NORMAL
Aspect ratio	HIGH	LOW	NORMAL
Inflection points	LOW	NORMAL	HIGH
Intensity variance	LOW	LOW	HIGH

Once the clusters are defined and the features from each connected component of each slice are extracted, the k‐means is applied until convergence is reached, resulting in the classification of fibroglandular tissue and blood vessels. To avoid the convergence in a local optimum, the process is repeated 20 times, each time with a different random initialization, and the chosen final classification is the one that gives the lowest value of the k‐means cost function.

This method is applied slice‐by‐slice rather than fully in three dimensions due to some blood vessels actually joining the fibroglandular tissue at a certain moment. If classification were done in three dimensions, these vessels would not be considered as separated components, and misclassification may occur.

**Figure 7:**
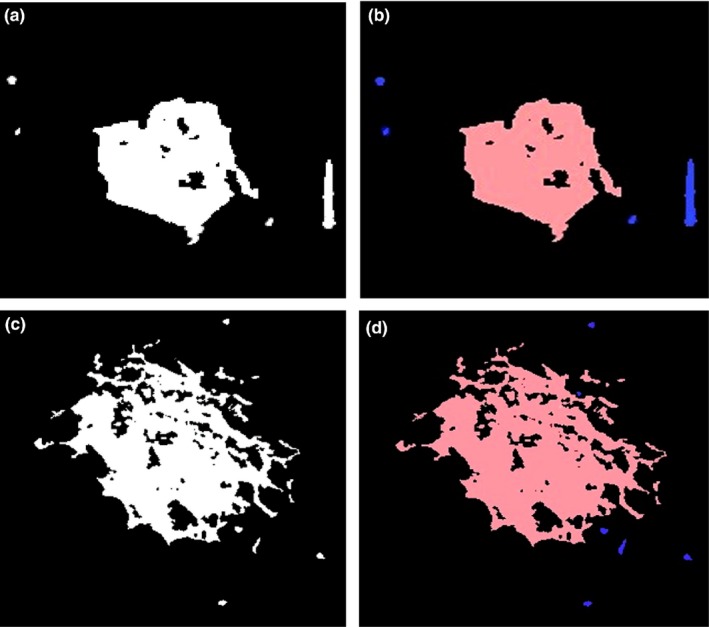
Example of the result of the cluster analysis (fibroglandular tissue, vasculature): (a, c) before and (b, d) after classification. [Color figure can be viewed at http://wileyonlinelibrary.com]

An example of the result of the cluster analysis for two slices is shown in Fig. 7.

After the k‐means classification of the last breast CT slice is finished, a continuity criterion for blood vessels is applied in the third dimension. This is the only step of the algorithm that works in full 3D dimensions. A 26‐connected 3D kernel is generated around each voxel classified as vasculature. The considered voxel is segmented as vasculature if at least three of the first nine positions or at least three of the last positions of the kernel are occupied by other voxels that were previously classified as vessels by the k‐means. Otherwise, that voxel is not classified as vasculature, but it is instead included in the fibroglandular segmentation. This simple criterion is aimed at segmenting as blood vessels only the connected components that actually have a continuity in the third dimension, therefore correcting small errors in fibroglandular classification by detecting small components of fibroglandular tissue that were previously misclassified by the clustering algorithm.

As a result, every voxel in the image has been classified as skin, fibroglandular, or adipose tissue, or vasculature.

### 2.C. Classification evaluation

To evaluate the results of the automatic segmentation, manual segmentation performed by an experienced breast radiologist was used as the gold standard. Three coronal slices (one near the nipple, one in the center of the breast and one near the chest) from 15 breast CT patient scans from each of the two breast CT systems were manually segmented, resulting in a total of 90 slices.

The performance of the algorithm was determined in terms of five similarity metrics.

- The Dice similarity coefficient (DSC), given by the following formula:

(11) $$DSC=\frac{2\cdot \left|A\cap B\right|}{\left|A\right|+\left|B\right|}$$

where A and B are the two samples (i.e., manually and automatically segmented images). The DSC ranges between 0 (the two samples are completely uncorrelated) and 1 (complete overlap of the two samples).

- The sensitivity, according to the following equation:

(12) $$Sensitivity=\frac{\mathrm{TP}}{TP+FN}$$

where TP is the number of true positives and FN the number of false negatives. Specificity was not used, since it is not an appropriate error metric for evaluating a segmentation result, due to its high sensitivity to the size of the evaluated object.

- The conformity coefficient,^36^ defined as:

(13) $$K_{c}=1-\frac{FP+FN}{\mathrm{TP}}$$

which can vary within a much wider range (−∞,1], providing a stricter evaluation of all our comparisons. A zero score is obtained when the number of correctly segmented voxels equals the number of mis‐segmented voxels, while it becomes −∞ in case the two segmented images (ground truth and processed) have no overlap. A score equal to 1 indicates a perfect overlap between the two segmented images.

- The traditional Hausdorff distance (basically the longest of the shortest distances between the two samples), defined as follows:

(14) $$HD=max\left\{\begin{array}{c} sup_{a\in A}inf_{b\in B}\left[d\left(a,b\right)\right],\\ sup_{b\in B}inf_{a\in A}\left[d(a,b)\right] \end{array}\right\}$$

where *sup* and *inf* refer to the supremum and infimum (which in a discrete case are equal to maximum and minimum) of the two compared datasets.

- Since the Hausdorff distance is very sensitive to outliers,^37^ an average distance (AVD) is also used, in which both suprema/maxima are replaced with the average operator.

The robustness of the algorithm to image noise was also evaluated by determining the impact on the five performance metrics listed above when re‐classifying the images after corrupting them with additional noise. For this, four levels of Gaussian noise (with zero mean and standard deviation varying from 0.01 to 0.04) were added to 15 slices, one from each patient image taken from the first dataset. The original slices are here considered the gold standard.

Finally, for further evaluation of the blood vessels classification, three different complete pre‐ and postcontrast injection patient breast CT images (acquired with the Koning breast CT system) were classified and compared. For this comparison, DSC and sensitivity were calculated in three dimensions between each pre‐ and postcontrast injection 3D breast CT image.

Beside these evaluations on the global performance of the algorithm, we also individually evaluated the methods in comparison to previously proposed works. We calculated the DSC (between manual and automatic segmentation) for the previously proposed methods and for our approach for ten breast CT slices. For skin classification, our algorithm was compared to the previously proposed morphological,^8^, ^10^ derivative^7^ and histogram‐based region‐growing^9^ methods. For fibroglandular tissue–adipose discrimination, our methods were compared to the previous histogram‐based region‐growing,^9^ Gaussian kernel‐based fuzzy C‐means,^7^, ^10^ and pixelwise support vector machine (SVM)^8^ approaches.

## 3 Results

**Figure 8:**
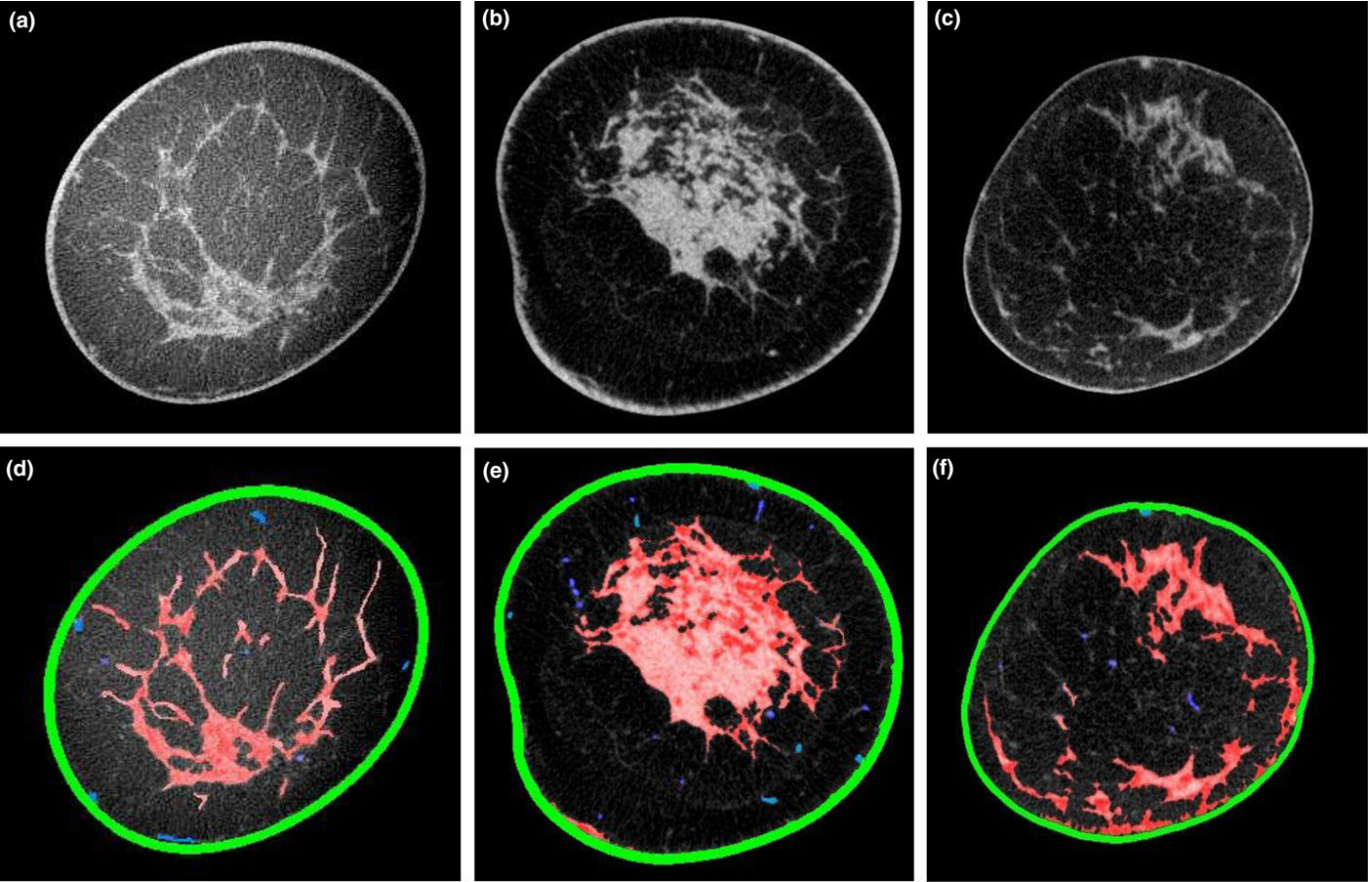
Three examples of (a, b, and c) original (coronal) slices from different breast CT patient images, and (d, e, and f) respective results of the automatic tissue classification, showing the skin, the fibroglandular tissue, the vasculature, and the adipose tissue. [Color figure can be viewed at http://wileyonlinelibrary.com]

**Figure 9:**
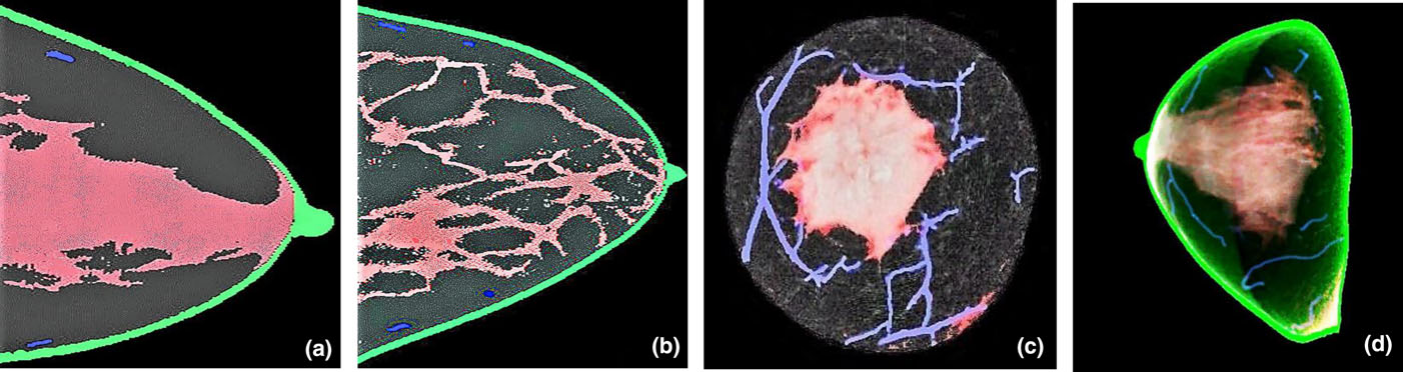
Two examples (a) and (b) of segmented breast CT slices in sagittal view, and two examples (c) and (d) of 3D renderings of an entire BCT image: (c) coronal view with the skin removed for an improved visualization and (d) sagittal view. [Color figure can be viewed at http://wileyonlinelibrary.com]

Figures 8 and 9 show some examples of the automatic segmentation.

**Figure 10:**
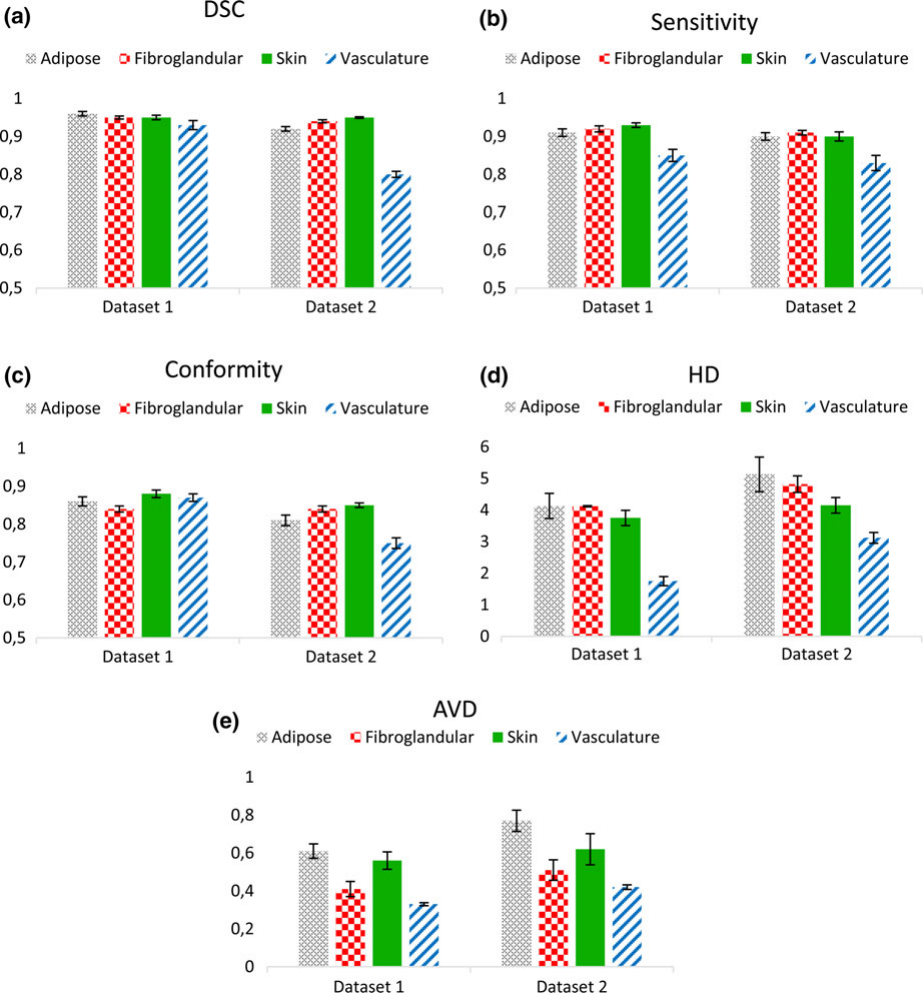
Results of the evaluations of the accuracy of algorithm. (a) Dice similarity coefficients, (b) sensitivity, (c) conformity coefficient, (d) Hausdorff Distance, and (e) average Hausdorff distance for the adipose, fibroglandular, skin, and vasculature between the results of the algorithm and manual segmentation. [Color figure can be viewed at http://wileyonlinelibrary.com]

**Figure 11:**
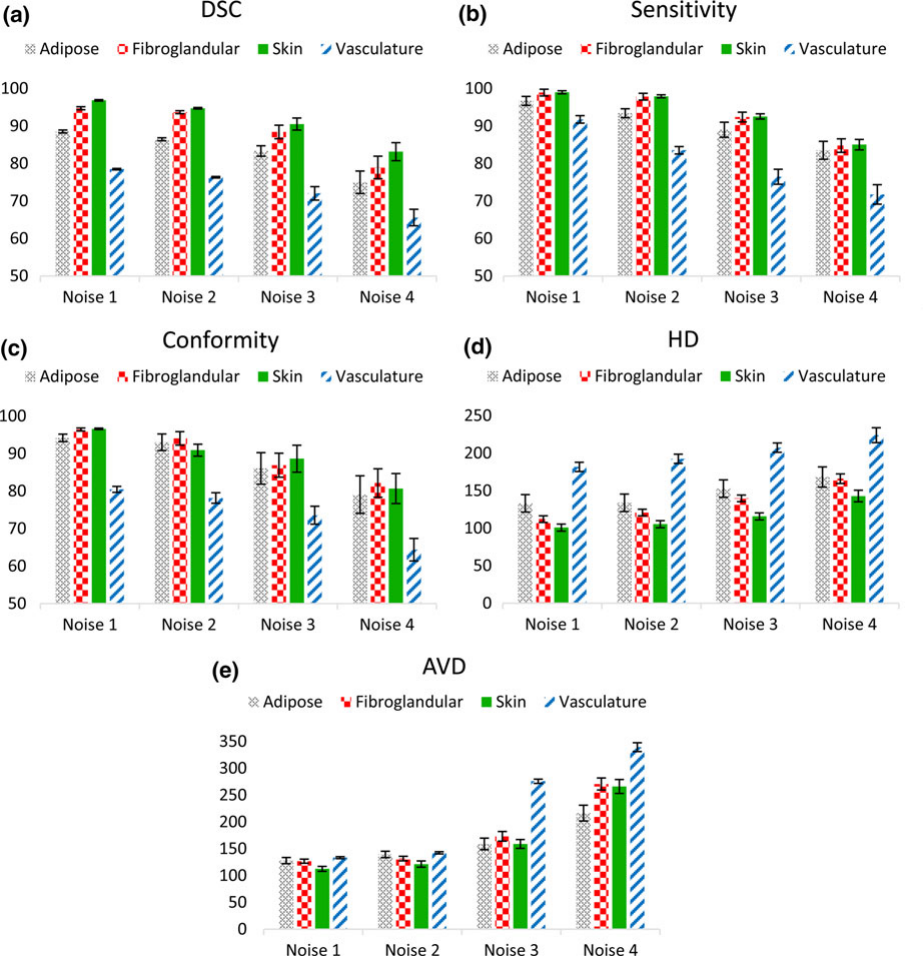
Results of the evaluations of the robustness of algorithm. (a) Dice similarity coefficients, (b) sensitivity, (c) conformity coefficient, (d) Hausdorff Distance, and (e) average Hausdorff distance for the adipose, fibroglandular, skin, and vasculature between manual segmentation and the result of the algorithm, after adding image noise (zero mean, standard deviation equal to 0.01, 0.02, 0.03, and 0.04). All metrics are here given as the ratio of the performance for the noisy images to that of the original images, in percentage. [Color figure can be viewed at http://wileyonlinelibrary.com]

**Figure 12:**
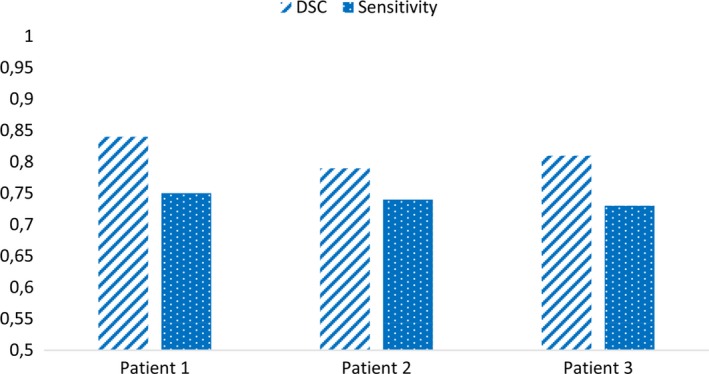
Dice similarity coefficients and sensitivity (calculated in three dimensions) for the automatic vasculature segmentation between three fully complete pre‐ and postcontrast injection images. The postcontrast images are considered the gold standard. [Color figure can be viewed at http://wileyonlinelibrary.com]

Figure 10 shows the average values with the standard error (given by the standard deviation divided by the square root of the sample size) of all similarity metrics for the 15 subjects (three slices each) between the manual and automatic segmentation for each of the two patient datasets, respectively. The results of the evaluation of the robustness to image noise (15 slices taken from the first dataset) are shown in Fig. 11, with the vertical axis being the ratio of the metric at the noise level and the metric at original (noise‐free) level, in percentage. The results of the comparison between pre‐ and postcontrast injection are displayed in Fig. 12 (postcontrast images are here considered the gold standard). These results are also presented in a tabular form in Table 2 (Appendix).

The algorithm proved to be robust to image noise and also reasonably accurate in blood vessels detection, even in unenhanced images. For all three patient images acquired pre‐ and postcontrast injection, the sensitivity presents a minimum value of 0.73. The false negative rate of 0.27 is due to the fact that a few major blood vessels (i.e., the ones detectable given the spatial resolution of the device) can be detected only with contrast enhanced imaging. In fact, if the manual segmentation of postcontrast injection images is considered the ground truth, the comparison between manual and automatic segmentation of the same precontrast injection images resulted in an average vasculature DSC of 0.780 ± 0.079 (manual segmentation, postcontrast vs manual segmentation, and precontrast) and 0.772 ± 0.081 (manual segmentation, postcontrast vs automatic segmentation, and precontrast), respectively. Finally, the average vasculature DSC between manual and automatic segmentation was 0.914 ± 0.027 for precontrast and 0.909 ± 0.018 for postcontrast injection images.

**Figure 13:**
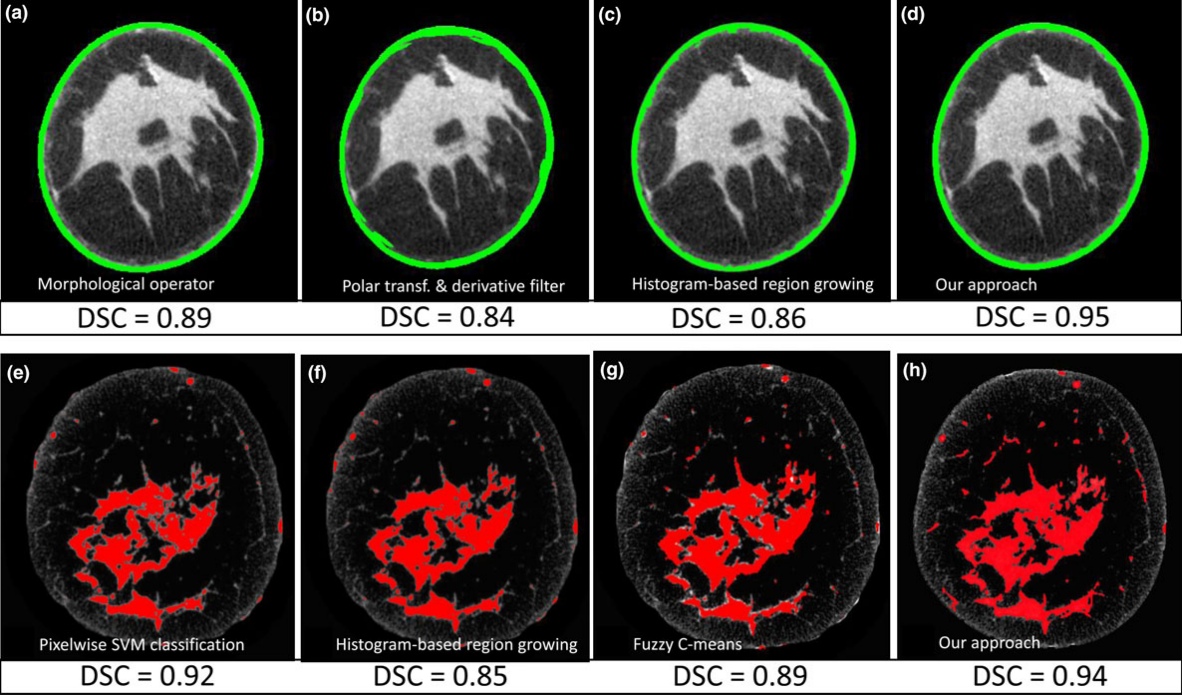
Example images of the performance of different algorithms in (a–d) breast skin and (e–h) fibroglandular tissue classification. Skin segmentation: (a) morphological operators‐based, (b) polar transformation and first order derivative filter, (c) histogram‐based region growing, and (d) our approach. Fibroglandular tissue segmentation: (e) SVM pixelwise classification, (f) histogram‐based region growing, (g) Gaussian kernel‐based fuzzy c‐means, and (h) our approach. [Color figure can be viewed at http://wileyonlinelibrary.com]

Finally, results of the evaluation of previously proposed methods for breast CT tissue classification are reported in Table 3, with some example images shown in Fig. 13 (Appendix).

Classification time was approximately 50 s per slice, although the computation time depends on the number of breast voxels per slice to be processed (fewer near the nipple, more near the chest), taking approximately 3–5 h for a complete volume.

## 4 Discussion

The proposed algorithm for breast CT image classification has been shown to result in accurate identification of the different tissue types, including blood vessels in unenhanced images, and is robust to image noise.

There are already a few algorithms in literature for breast CT image segmentation.^7^, ^8^, ^9^, ^10^ For each algorithm, there are two major steps: one for classifying the skin, and the other for the two major breast tissue types (adipose and fibroglandular tissue).

Some proposed methods for skin segmentation only rely on appropriate boundary detection, which could fail when other structures, such as blood vessels or spots of fibroglandular tissue, are located close to the interior edge of the skin. Other skin segmentation methods use a morphological operator with constant dimensions based on the average breast skin thickness reported in literature, therefore not taking into account interpatient variability.

Regarding the fibroglandular‐adipose tissue discrimination, some algorithms require the setting of some parameter values according to some image properties (e.g., ring artifacts), making the algorithms not completely automatic. In other algorithms, the use of supervised methods results in a performance that might change according to the training dataset, especially when classifying images acquired with different breast CT systems. Methods based on global information, such as the image histogram, only may lead to challenges in those slices that experience nonuniformities that affect the histogram threshold on a global basis (e.g., due to artifacts and noise). By evaluating these methods on our dataset we encountered, in some cases, the limitations stated above related to inaccuracies in the classification of the skin boundaries, parameter tuning, sensitivity to noise, and long processing time. Finally, all algorithms divide the breast into three main tissue types (skin, adipose, and fibroglandular) without considering blood vessels separately. This means that blood vessel voxels are considered either as adipose tissue or as fibroglandular tissue, leading to an overestimation of one of these tissue types. These limitations affected the performance of the previous algorithms during our comparison with the algorithm proposed here.

The differences in the performance of our algorithm across the two data sets is mainly due to the lower dose (comparable with a two‐view traditional mammogram) used for the UC Davis images (dataset 2), resulting in lower contrast resolution and increased noise. These factors especially affect the blood vessel segmentation the most, making their detection more challenging in noisy low‐contrast images due to their small size, low intensity, and unpredictability in localization, which is also apparent from the analysis of the degraded images from the first dataset. Moreover, results from pre‐ and postcontrast images show that, in unenhanced images, the algorithm fails in the detection of blood vessels that result in very low HU voxel values (or that lay within the fibroglandular tissue, resulting in a too low contrast). Similarly, at least for the dataset used for our validation, the high vasculature DSCs between manual and automatic segmentation underline the difficulty of this task, even for a radiologist.

Results from all similarity metrics evaluated in this work were satisfying and consistent. Sensitivity resulted in similar results to the DSC; the conformity coefficient, as expected, resulted in slightly lower values compared to DSC, and the two shape‐based metrics (HD and AVD) showed to increase as the noise content becomes larger. In any case, the highest distances (in voxels) resulting from these latter two metrics are 5.2 and 0.77, respectively, for HD and AVD. The difference in these two results is not surprising, since the HD is a measure of the longest (out of the shortest) distance between the two compared objects (i.e., it could reach very high values even if the two objects are, on average, well registered). This is the reason why the AVD is also included. By averaging all the possible distances between the compared objects, a more reliable and less outlier‐sensitive measure is obtained.

The high accuracy of the proposed method can be attributed to the use of different image analysis techniques for classifying each tissue type. Each technique proved to be particularly suitable for a certain type of tissue. For instance, breast skin segmentation proved to have high performance due to the combination of intensity‐ and region‐based criteria. The former are useful to differentiate the skin from the adjacent adipose tissue, while the latter avoid misclassification of voxels that actually belong to blood vessels or fibroglandular tissue which are located close to the skin. Using only intensity‐based criteria would lead to over‐classification, while using only geometrical and topological operators would not be robust enough, since the skin may appear with different shapes and locations depending on the distance from the nipple.

**Figure 14:**
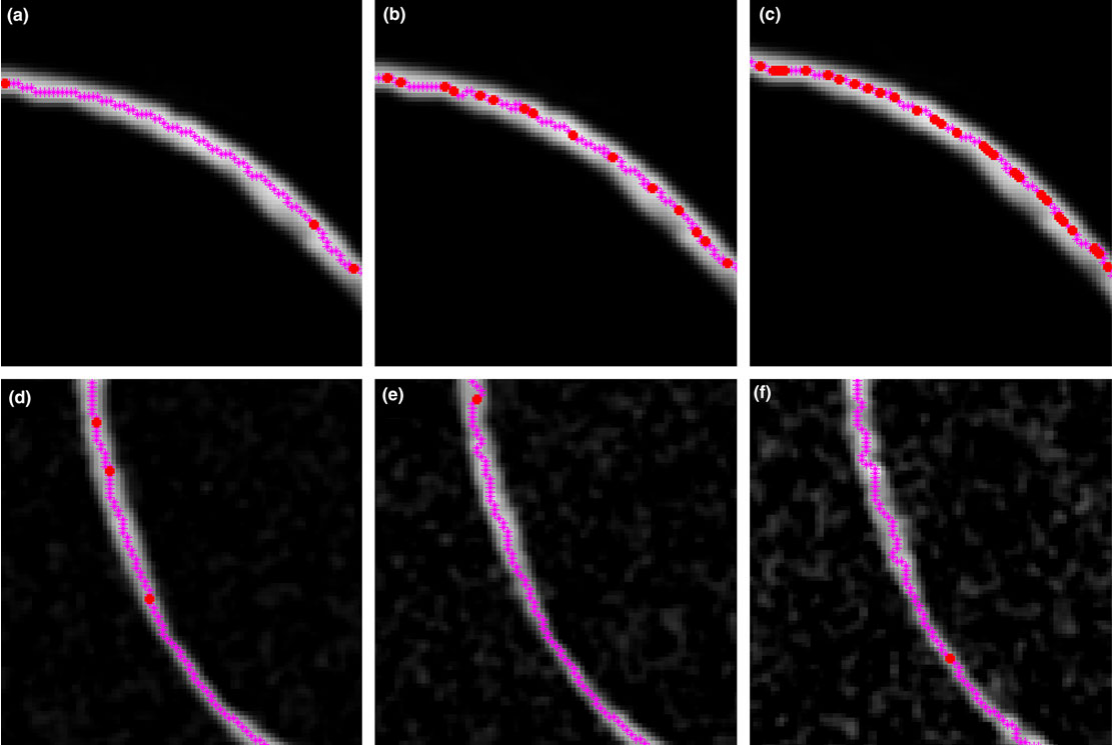
Example of results of the centerline extraction method on a phantom image with varying conditions: tolerance‐to‐zero threshold equal to (a) 10^−6^, (b) 10^−5^, and (c) 10^−3^; Gaussian noise with zero mean and standard deviation equal to (d) 0.02, (e) 0.04, and (f) 0.08. [Color figure can be viewed at http://wileyonlinelibrary.com]

**Figure 15:**
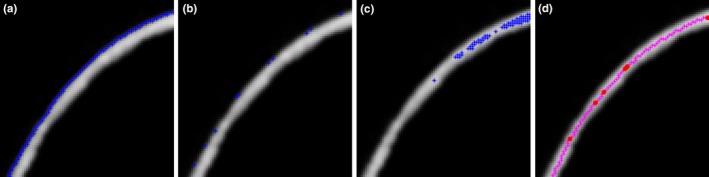
Example of the robustness of the centerline extraction method on a phantom image to position and number of initial seed points. Panels (a), (b), and (c) show different seed points initializations, while panel (d) the resulting centerline, equal for the three initial settings. [Color figure can be viewed at http://wileyonlinelibrary.com]

**Figure 16:**
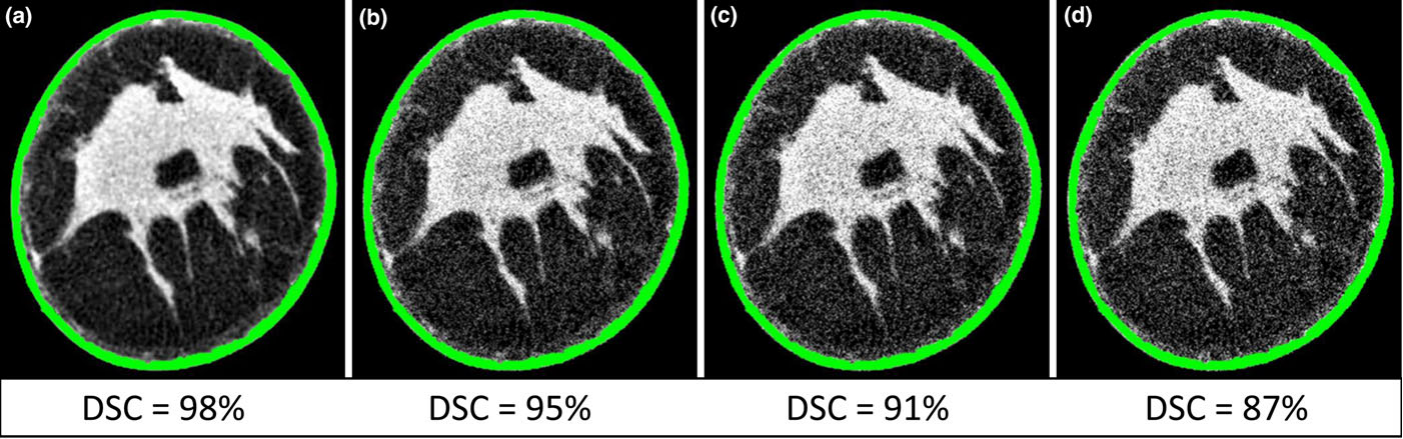
Robustness of the region growing for skin segmentation to image noise with different standard deviations: (a) 0.01, (b) 0.02, (c) 0.03, and (d) 0.04. The DSCs are given as a percentage of the DSC of the noise‐free image. [Color figure can be viewed at http://wileyonlinelibrary.com]

To assess the performance of the region‐growing method for skin segmentation, which strongly depends on the previous centerline extraction from the skin layer, we evaluated the robustness of the centerline detection scheme to initialization, noise and *tolerance‐to‐zero* condition. In previous methods, the ridge detection step has always been critical in the extraction of a smooth and connected centerline. Regarding the initialization step, in traditional approaches, it basically depends on the tolerance for equal‐to‐zero condition in the product between the image gradient and the N‐1 eigenvectors, on the position of seed points and on the scale at which the intensity, gradient, and Hessian of the image are calculated. For this, we decided to keep these values constant for the whole model (unit scale, and tolerance‐to‐zero equal to 10^−6^). This allows to always find at least a few points of the ridge, which will be then connected through the subsequent intensity‐based search algorithm. Regarding the seeds position (which, in our application, is defined on the outer edge of the skin), we tested the algorithm by randomly changing their location within the skin layer and by decreasing their number. All experiments led to same final centerline detected. Regarding the robustness to image noise, the intensity‐based search algorithm could overcome the local discontinuities and produce a fully connected centerline, enabling the introduction of the constraints needed to extract the skin layer with the subsequent region growing. Importantly, the subsequent intensity‐based search connecting all ridge points together allows for avoiding the need for user‐selectable parameters during the centerline extraction. Examples of the centerline resulting from varying the *tolerance‐to‐zero* threshold, the noise content and the initial number and location of seed points are shown in Figs. 14 and 15 of the Appendix, respectively. The accuracy of the centerline‐based region growing related to image noise is shown in Fig. 16 (Appendix).

**Figure 17:**
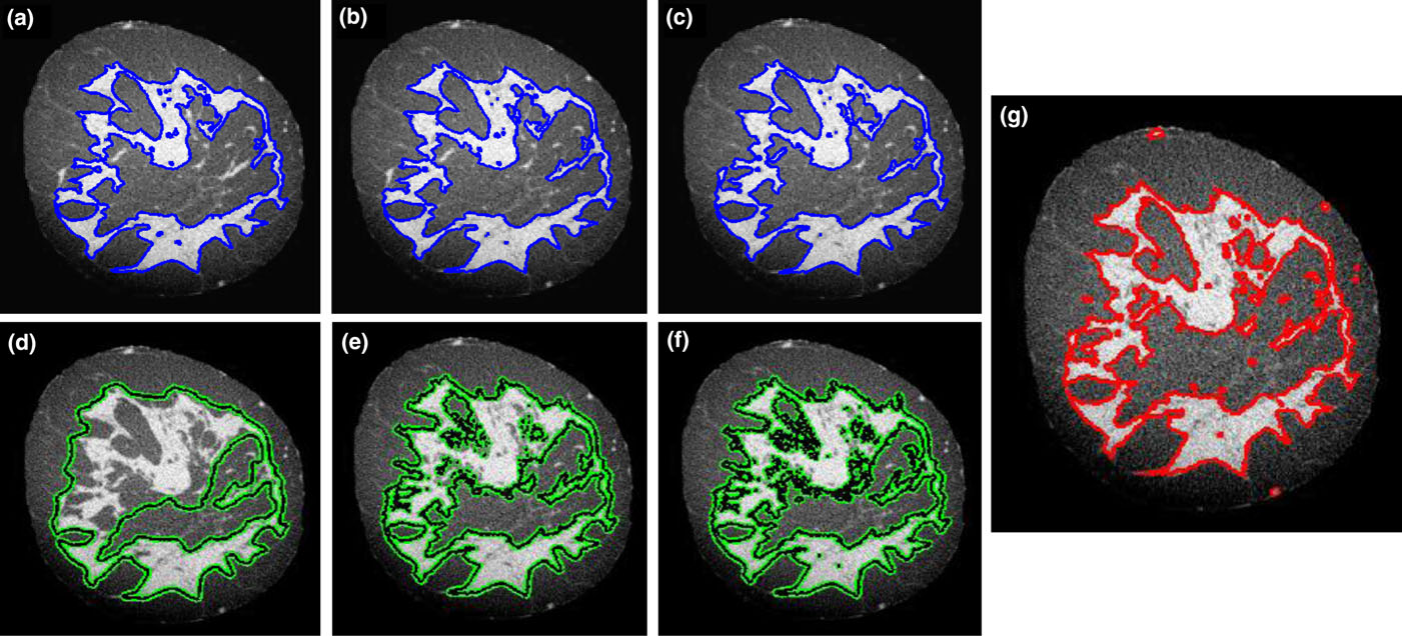
Example of three active contour models in fibroglandular tissue segmentation. Panels (a), (b), and (c) display the traditional localized active contour for three different radii of the characteristic function: (a) 9 voxels, (b) 15 voxels, and (c) 25 voxels. Panels (d), (e), and (f) display the traditional active contour without edges for three different weights of the smoothing term: (d) 10^−1^, (e) 10^−2^, and (f) 10^−3^. Panel (g) shows the result of the energy minimizing framework proposed in this work. [Color figure can be viewed at http://wileyonlinelibrary.com]

To better evaluate the effectiveness of the active contour method implemented in this work, we compared our method with some previously defined models: geodesic contours,^19^ contours without edges,^20^ and localized active contours.^21^ Geodesic active contours are very sensitive to image inhomogeneities, and depend on initialization. Given the non‐negligible noise content of breast CT images, and difficulty to automatically initialize the contour close to the boundary of the object, we could not achieve any acceptable results by applying these contours. Regarding the region‐based active contour without edges (Chan‐Vese), the main limitation is the setting of the weight of the smoothing term, since it has to be adjusted each time according to the SNR of the image. Its choice may strongly affect the segmentation result, since a noncorrect setting of the smoothing term can lead to over‐segmentation. Finally, in localized active contours, by restricting the region energy computation to a small neighborhood of contour points using a characteristic function, the setting of the smoothing weight is less critical. However, the radius of the characteristic function used to evaluate the voxel values inside and outside the contour still has to be set according to the image noise and appearance. Smaller values usually achieve a more precise object contouring, but may also lead to under‐segmentation (the opposite may happen for values that are too large). Some example images of the different contour models evaluated are shown in Fig. 17 (Appendix).

The active contour presented in this work is able to overcome some limitations of traditional edge‐based models (e.g., it avoids local minima), but still including edge information which is useful to correctly identify fibroglandular tissue and vessels boundaries. Furthermore, the other region‐based contributions (mean and variance) make it robust to image noise, and the global energy does not require any user‐selectable weight. Overall, the proposed contour could well discriminate the adipose tissue from remaining structures. However, as the noise content increases, tissues edges become more blurred, affecting negatively the exact matching of the contour model and the real object boundaries.

Finally, the unsupervised clustering algorithm showed to be robust in recognizing blood vessels well even in the case of branches or irregularities (still quite uncommon within one slice).

Blood vessel segmentation has been extensively studied in literature,^38^ but applying one of the already implemented methods in unenhanced breast CT images may lead to a few limitations. First, blood vessels appear just as several spots among many other structures (such as fibroglandular tissue), and have a high level of spatial unpredictability. Moreover, without using a contrast agent they have the same x‐ray attenuation as skin and fibroglandular tissue, resulting in very similar Hounsfield unit values. Furthermore, due to the low dose levels used (especially for the second dataset), images are noisy. These factors make the use of intensity‐based methods or extraction schemes difficult to apply. It would also be difficult to use centerlines and region‐based methods for recognizing blood vessels in breast CT images, since it would be hard to automatically define the seed points or the region boundaries without including other structures (such as fibroglandular tissue). Therefore, the method implemented here mostly considers geometrical and shape‐related features which can represent the two tissue types (vessels and fibroglandular tissue) well, and overcomes the difficulties mentioned above. Moreover, using the whole breast CT image allows to overcome the difficulty of defining the number of clusters within a single slice, resulting in a correct classification even in those slices which actually contain only one tissue type (this could happen especially in slices near the nipple, where only fibroglandular tissue might be present).

Finally, the continuity criterion adds three‐dimensional information which allows to correct small errors that may have occurred in the first classification.

The robustness of the algorithm was tested with four different noise levels with increasing standard deviation. The increase in noise resulted in corrupted images with a signal‐to‐noise ratio (SNR) 5% to 30% lower than that of the images of the UC Davis dataset. To achieve a dose estimate, we scanned a breast CT phantom (Koning Corporation, 13 cm diameter – 11.5 cm length, with a Br20/80 background material equivalent to 20%/80% glandular‐adipose tissue) with different mA (constant 8 ms pulse) and measured the standard deviation in a homogeneous ROI for different mA. We then measured the standard deviation of a ROI in few homogenous areas of patient images (the ones corrupted with added Gaussian noise), and determined the mA levels that would give similar noise levels. We then calculated the dose for each added noise level. Although approximate, this process gave us estimations of the dose related to the noise content, which resulted approximately between 2.5 and 5 mGy.

**Figure 18:**
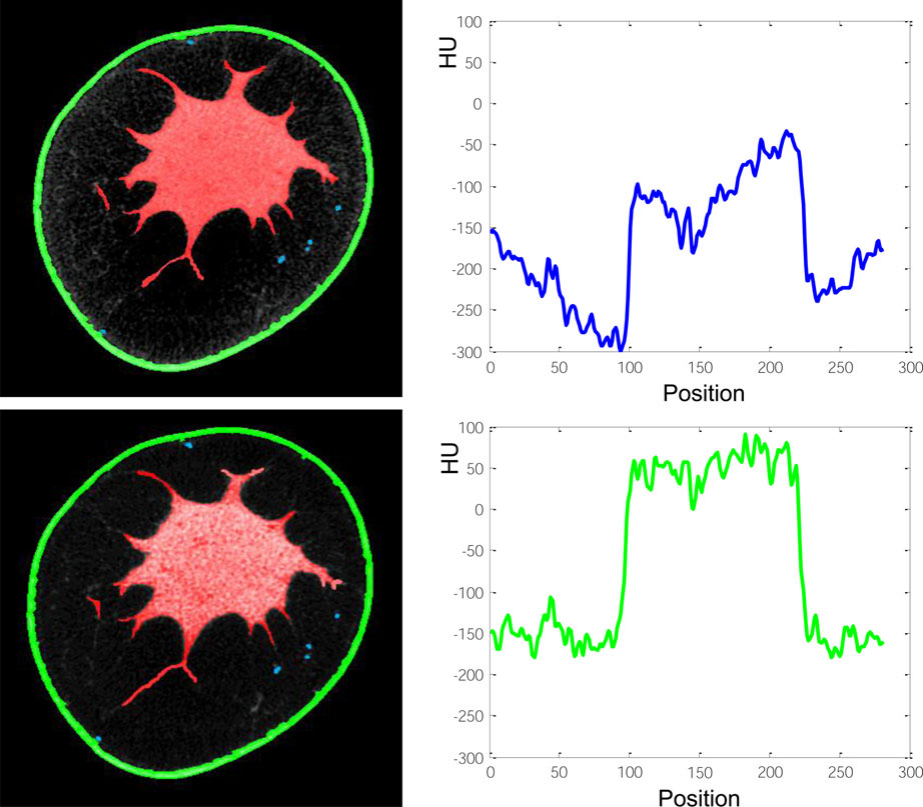
Segmented breast CT slice with (top panel) and without (bottom panel) cupping artifacts due to incomplete scatter correction, resulting in a mutual DSC of 97%. [Color figure can be viewed at http://wileyonlinelibrary.com]

The algorithm is also robust to image nonuniformities due to incomplete scatter correction. Cupping artifacts due to scatter do not affect its performance (as shown in Fig. 18 of the Appendix), since none of the methods are simply intensity‐based but include different morphological, regional and mathematical criteria. The algorithm does not take into account the classification of the pectoralis muscle, which in some cases can be seen in a few posterior slices near the chest. Therefore, slices containing the pectoralis were removed from the classification. Overall, this should not affect the fibroglandular tissue percentage within the breast considerably, since the amount of fibroglandular voxels in slices containing the pectoralis is usually small.

Automatic pectoralis segmentation can be a challenging task, since its attenuation is very similar to fibroglandular tissue and since it often appears connected (along the sagittal direction) to other tissue types (especially fibroglandular tissue). A method to classify the pectoralis might be added in future work. In this case, supervised classification methods might be considered for an accurate detection.

In this study, we used manual segmentation as the gold standard because we used data from patients. Although manual segmentation is very time consuming, with greater numbers of slices manually segmented in each patient further investigation of the algorithm could be achieved.

The algorithm (except for the continuity criterion for vasculature postprocessing) works in two dimensions by processing the breast CT images in a slice‐by‐slice manner. It is still to be proven whether a fully 3D algorithm could lead to advantages in the segmentation (also because the HU values tend to differ among the slices according to the distance from the nipple, due to the increasing amount of breast voxels toward the chest). The ability to fully incorporate the third dimension might provide further information for detecting thin low‐intensity components that could otherwise be considered noise, but it would drastically increase the computational cost.

The algorithm was implemented in MATLAB (The MathWorks, Natick, MA, USA), and therefore could be speeded up by using lower level languages like C++ and/or parallel processing, especially using Graphic Processing Units (GPU).

Applications of the proposed algorithm in breast imaging research can be divided into three main groups. First, the evaluation of breast density can be used for dosimetry evaluation and simulation‐based radiation dose analysis. In fact, the 3D glandularity patterns provided by the segmentation algorithm could be used as input in Monte Carlo simulations to obtain a map of radiation dose throughout a real breast. This would be useful to better define radiation dose guidelines not only for breast CT but also for other x‐ray‐based breast imaging techniques (e.g., mammography). Second, the algorithm could be useful in the design of realistic patient data‐based phantoms, which could be used for optimizing the acquisition process as well as breast CT image reconstruction and analysis methods.

Lastly, the present work can be used for tissue pattern characterization and quantification to extract biomarkers related to breast cancer development. Examples include breast density quantification (i.e., quantification of breast glandularity), automatic identification of breast skin thickening, and monitoring of blood vessels maturation and growth, which are all factors that seem to be related to breast diseases and cancer development.

## 5 Conclusions

The proposed algorithm for breast CT image segmentation resulted in accurate and robust classification of breast tissues (fibroglandular and adipose tissue, skin, and vasculature) with no prior training or threshold setting. It can be used for 3D breast density quantification and tissue pattern characterization, both biomarkers of cancer development, realistic patient‐based radiation dose analysis and development of patient image‐based phantoms which could be used for breast imaging research.

## Conflicts of interest

The authors have no relevant conflicts of interest to disclose.

## Appendix

**Table 2:** All quantitative results for the two image datasets and for the four datasets corrupted with Gaussian noise (in four steps, with increasing standard deviation from 0.01 to 0.04). All results are in comparison with the same manually segmented image dataset

Dataset	Tissue type	DSC	Sensitivity	Conformity	HD	AVD
Original 1	Adipose	0.96 ± 0.03	0.91 ± 0.05	0.86 ± 0.06	4.13 ± 1.99	0.61 ± 0.19
	Fibroglandular	0.95 ± 0.02	0.92 ± 0.04	0.84 ± 0.04	4.12 ± 0.10	0.41 ± 0.20
	Skin	0.95 ± 0.03	0.93 ± 0.03	0.88 ± 0.05	3.75 ± 1.21	0.56 ± 0.23
	Vasculature	0.93 ± 0.06	0.85 ± 0.08	0.87 ± 0.05	1.75 ± 0.72	0.33 ± 0.04
Original 2	Adipose	0.92 ± 0.03	0.90 ± 0.05	0.81 ± 0.07	5.13 ± 2.74	0.77 ± 0.28
	Fibroglandular	0.94 ± 0.02	0.91 ± 0.03	0.84 ± 0.04	4.82 ± 1.31	0.51 ± 0.27
	Skin	0.95 ± 0.01	0.90 ± 0.06	0.85 ± 0.03	4.15 ± 1.23	0.62 ± 0.41
	Vasculature	0.80 ± 0.04	0.83 ± 0.10	0.75 ± 0.07	3.12 ± 0.85	0.42 ± 0.06
Noise 1	Adipose	0.85 ± 0.02	0.88 ± 0.06	0.81 ± 0.05	5.50 ± 2.41	0.78 ± 0.29
	Fibroglandular	0.90 ± 0.02	0.91 ± 0.09	0.81 ± 0.02	4.62 ± 0.91	0.52 ± 0.19
	Skin	0.92 ± 0.01	0.93 ± 0.04	0.85 ± 0.01	3.78 ± 0.88	0.63 ± 0.23
	Vasculature	0.73 ± 0.01	0.78 ± 0.05	0.70 ± 0.04	3.18 ± 0.54	0.44 ± 0.09
Noise 2	Adipose	0.83 ± 0.02	0.85 ± 0.06	0.80 ± 0.11	5.53 ± 2.41	0.85 ± 0.30
	Fibroglandular	0.89 ± 0.02	0.90 ± 0.09	0.79 ± 0.09	4.98 ± 0.91	0.54 ± 0.21
	Skin	0.90 ± 0.01	0.92 ± 0.04	0.80 ± 0.08	3.95 ± 0.88	0.68 ± 0.29
	Vasculature	0.71 ± 0.01	0.71 ± 0.05	0.68 ± 0.07	3.37 ± 0.54	0.47 ± 0.10
Noise 3	Adipose	0.80 ± 0.07	0.81 ± 0.10	0.74 ± 0.21	6.31 ± 2.41	0.97 ± 0.54
	Fibroglandular	0.84 ± 0.09	0.85 ± 0.13	0.73 ± 0.16	5.76 ± 0.91	0.71 ± 0.45
	Skin	0.86 ± 0.08	0.87 ± 0.07	0.78 ± 0.18	4.34 ± 0.88	0.89 ± 0.41
	Vasculature	0.67 ± 0.09	0.65 ± 0.10	0.64 ± 0.12	3.63 ± 0.54	0.91 ± 0.20
Noise 4	Adipose	0.72 ± 0.15	0.76 ± 0.12	0.68 ± 0.25	6.95 ± 2.78	1.32 ± 0.74
	Fibroglandular	0.75 ± 0.15	0.78 ± 0.18	0.69 ± 0.19	6.84 ± 1.31	1.11 ± 0.56
	Skin	0.79 ± 0.12	0.80 ± 0.14	0.71 ± 0.20	5.36 ± 1.45	1.49 ± 0.65
	Vasculature	0.61 ± 0.11	0.61 ± 0.13	0.56 ± 0.15	3.92 ± 0.87	1.12 ± 0.41

**Table 3:** Dice similarity coefficients of all the tested algorithms for breast tissue classification against manual segmentation

Tissue type	Method	Ref.	DSC
Skin	Polar transformation and derivative filter	7	0.84 ± 0.07
	Morphological operators‐based	8, 10	0.89 ± 0.08
	Histogram‐based region growing	9	0.86 ± 0.04
	Proposed algorithm	–	0.95 ± 0.05
Fibroglandular — Adipose	Histogram‐based region growing	9	0.84 ± 0.08
	Gaussian kernel‐based fuzzy C‐means	7, 10	0.89 ± 0.09
	Pixelwise SVM‐based classification	8	0.92 ± 0.05
	Proposed algorithm	–	0.94 ± 0.03
